# Klippel–Feil syndrome revealed by post-traumatic neck pain: Case report and literature review

**DOI:** 10.1016/j.radcr.2025.07.056

**Published:** 2025-08-23

**Authors:** Salma El Kadiri, Ibtissam El Bqaq, Hind Qajia, Zyneb Izi, Latifa Chat, Mohammed Jiddane, Firdaous Touarsa, Siham El Haddad

**Affiliations:** a Pediatric Radiology Department, Mother and Child Hospital, Rabat, Morocco; b Neuroradiology Service, Specialty Hospital, Rabat, Morocco

**Keywords:** Klippel–Feil syndrome (KFS), Brain trauma, Cervical pain

## Abstract

Klippel–Feil syndrome (KFS) is a congenital anomaly involving the fusion of two or more cervical vertebrae. It is characterized by a low hairline at the nape of the neck, a short neck, and a limited range of motion. Here, we present the case of a 10-year-old girl who suffered brain trauma in a motor vehicle accident and subsequently experienced persistent occipital headaches and dizziness. A brain computed tomography scan performed to evaluate these symptoms revealed incomplete fusion of two cervical vertebrae (C2-C3), leading to a diagnosis of KFS. Magnetic resonance imaging of the spine was conducted to investigate potential spinal abnormalities. In this article, we discuss the pathogenesis, associated abnormalities, subtypes, complications, and differential diagnosis of KFS. There is no cure for KFS. Treatment requires a multidisciplinary approach involving neurologists, orthopedic surgeons, pediatricians, physical therapists, and neurosurgeons, depending on the severity of the symptoms.

## Introduction

Klippel–Feil syndrome (KFS) is a rare congenital condition characterized by aberrant segmentation or fusion of the cervical vertebrae during early embryogenesis. This leads to a shorter cervical spine. KFS, which was first characterized by Maurice Klippel and André Feil in the early twentieth century, is more common in females but unusual in the general population. The traditional clinical triad involves a short neck, a low posterior hairline, and limited cervical movement. However, this comprehensive presentation is only seen in a subset of cases. The syndrome has a wide range of severity, from asymptomatic to severely functionally impaired with accompanying anomalies.

Since its initial description, the syndrome has been linked to a variety of spinal and extraspinal abnormalities [1,2]. These abnormalities may lead to chronic headaches, restricted cervical mobility, and neck muscle soreness. Cervical spinal deformities, instability, spinal stenosis, and neurological impairments may also be present. Additional congenital anomalies are occasionally observed [3,4].

## Case report

A 10-year-old girl was referred to the pediatric emergency department for persistent occipital headaches and dizziness that were not relieved by analgesics. She had not experienced these symptoms prior to the accident, which occurred 2 months earlier, and they began gradually in the weeks following the accident. During the accident, she sustained a minor head injury and briefly lost consciousness. At that time, her clinical status was stable with no neurological deficits. Vital signs recorded in the emergency department immediately after the accident were normal, and a physical examination revealed only a mild head contusion.

Her past medical history was unremarkable, with no prior hospitalizations, chronic illnesses, or surgeries. Family history revealed no known genetic disorders or skeletal anomalies. However, a clinical examination confirmed her parents’ observation of a subtle shortening of her neck over time.

Upon admission, the patient reported severe occipital headaches and dizziness without associated visual disturbances or neurological deficits. Physical examination revealed cervical spine tenderness and restricted range of motion, particularly in lateral flexion and rotation. The neurological assessment was normal. There was no facial asymmetry, hearing loss, or limb deformities observed. Cardiovascular and respiratory examinations were unremarkable.

Laboratory findings were unremarkable. The hemoglobin level was 12.5 g/dL (normal range: 11.5-15.5 g/dL), the white blood cell count was 7200/mm³ (normal range: 5000-10,000/mm³), and the platelet count was 280,000/mm³ (normal range: 150,000-400,000/mm³). The C-reactive protein and erythrocyte sedimentation rate were within normal limits. Liver and renal function tests showed no abnormalities.

**Fig. 1:**
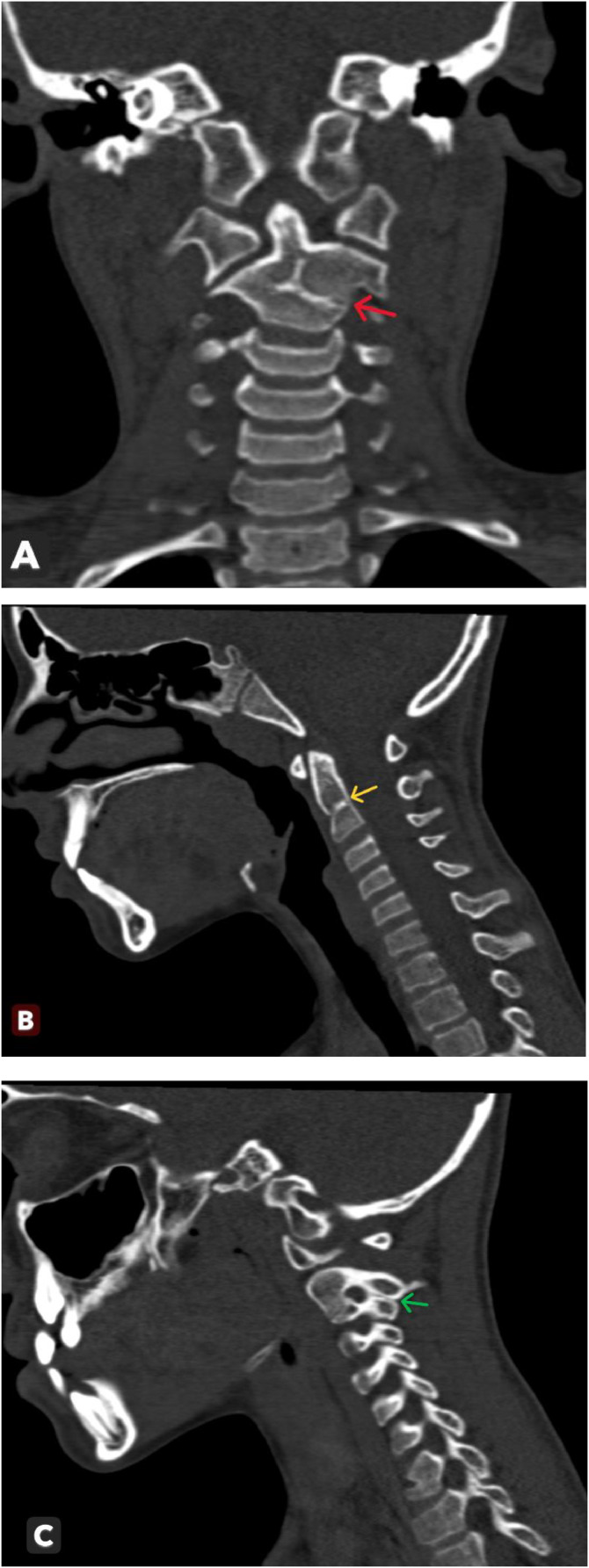
Coronal (A) and sagittal (B and C) computed tomography images of the cervical spine showing straightening of the cervical spine with fusion (block) of the C2-C3 vertebrae (red and yellow arrows), associated with two left transverse processes (green arrow).

A brain computed tomography scan was performed to check for intracranial complications. Although no abnormalities were found, the scan incidentally revealed a cervical spine malformation within the scanned region. A targeted cervical spine computed tomography scan confirmed the incomplete fusion of the second and third cervical vertebrae (C2-C3) and the presence of duplicated left transverse processes (Fig. 1).

**Fig. 2:**
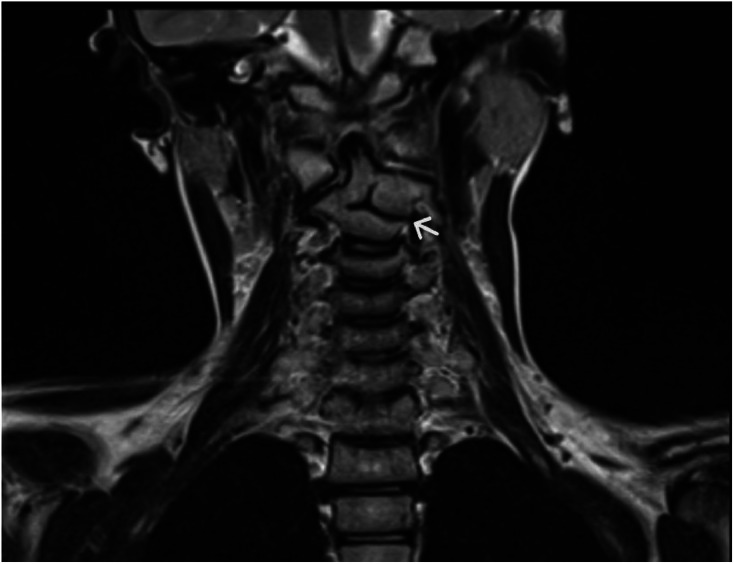
Coronal T2 magnetic resonance imaging (MRI) sequence confirming incomplete fusion of the C2 and C3 vertebral bodies, with deviation of the odontoid process to the right (white arrow).

Later, magnetic resonance imaging was used to assess the integrity of the spinal cord and detect any anomalies. The analysis ruled out spinal cord compression and related illnesses, such as Chiari malformations and diastematomyelia. No spinal abnormalities were discovered at the thoracolumbar levels. These findings led to the diagnosis of KFS Type I (Fig. 2).

Following the diagnosis, the patient was referred to orthopedic and neurosurgery teams for a multidisciplinary evaluation in order to devise an appropriate treatment plan. Conservative treatment was advised, which included regular clinical follow-ups and a tailored physiotherapy program aimed at enhancing cervical mobility and preventing future issues. During the brief follow-up period, the patient’s neurological condition was stable, with no new symptoms or functional impairment. Plans were made for ongoing monitoring to detect any potential progression of spinal abnormalities or neurological impairment, allowing for prompt intervention if necessary.

## Discussion

KFS is a congenital disorder first described by Klippel and Feil in 1912. It is caused by a failure of segmentation in one or more levels of the cervical spine. It may also involve segmentation anomalies in the thoracic and lumbar regions. The term encompasses various forms of congenital fusion anomalies of the cervical spine, regardless of their extent. KFS occurs in one out of every 40,000-42,000 newborns worldwide, with females accounting for approximately 60% of cases. Nevertheless, the actual prevalence of KFS may be underestimated due to variability in phenotypic expression and the absence of extensive, population-based screening studies. The pathogenesis of KFS remains uncertain, though mutations in the GDF6, GDF3, MEOX1, MYO18B, and RIPPLY2 genes have been associated with dominant and recessive forms of the disease. A recent genetic analysis identified five rare variants (BAZ1B, FREM2, VANGL1, SUFU, and KMT2D) associated with cervical fusion in patients with KFS [5].

Moreover, impaired segmentation and differentiation of cervical somites has been identified as a contributing factor in the pathogenesis of KFS. The clinical presentation of KFS is highly heterogeneous. The classic diagnostic triad includes a short neck, a low posterior hairline, and restricted cervical range of motion. However, recent studies have reported that nearly half of affected individuals do not exhibit these hallmark features, as observed in our patient. A more detailed analysis demonstrated that only patients with Type III KFS can present the complete clinical triad. A more detailed analysis demonstrated that only patients with Type III KFS present the complete clinical triad [5].

This variability was evident in our situation, where the patient had just a slightly shorter neck and restricted cervical movement. The diagnosis was made unintentionally in the setting of prolonged post-traumatic symptoms, with no typical clinical suspicion.

According to Menger et al. [6], approximately two-thirds of individuals with Klippel-Feil syndrome remain asymptomatic for several years. Among those who develop symptoms, patients commonly present with axial complaints such as neck pain and restricted mobility, while more complex deformities may predispose them to myelopathy and radiculopathy. This spectrum of clinical presentations aligns with our case, where axial symptoms were predominant without severe neurological impairment.

Beyond the variable phenotypic presentation characteristic of KFS, emerging research has revealed comparable heterogeneity in abnormalities affecting other organ systems. These abnormalities often represent the main reason patients seek medical attention. The National Institutes of Health and the National Organization for Rare Disorders report that clinical manifestations and symptoms of KFS include a wide range of osseous and nonosseous anomalies. Skeletal deformities, such as scoliosis or kyphosis, are seen in approximately 70% of cases [7]. Spina bifida occulta occurs in about 45% of cases, and craniovertebral junction anomalies are frequently observed as well. Additional skeletal abnormalities include Sprengel deformity (present in about 30% of patients), rib fusion or defects, syndactyly, diffuse hypoplasia of the upper limbs, and torticollis. Cosmetic concerns, including facial asymmetry and flattening of the neck, affect about 20% of individuals. Otolaryngologic manifestations, such as hearing loss (occurring in about 30% of cases), ptosis, and facial nerve palsy, have also been reported [8]. Furthermore, visceral anomalies are common. Congenital heart disease affects between 4% and 14% of patients, and genitourinary tract malformations are reported in up to 35%. Cardiovascular complications primarily consist of congenital heart defects, valvular disease, and vascular anomalies [9]. In our case, no associated anomalies were identified, highlighting the broad clinical spectrum of KFS and variability in systemic involvement.

According to the literature, congenital urogenital anomalies occur in 25%-35% of patients with KFS. The most frequently reported anomaly is unilateral renal agenesis [10].

A new analysis revealed that the most prevalent type of vertebral fusion in KFS is between C2 and C3. The second most common fusion is between C1 and C2, followed by fusions between C4 and C5. These results differ from those of previous studies, in which Type I KFS was the most common (46% of cases), followed by Types II and III (30% and 24%, respectively). Males were more likely to be classified as Type I, while females were more likely to be classified as Type II. Genetic mutations, particularly in the GDF3 and GDF6 genes, were reported; these mutations were mostly found in male patients with Type I KFS [9]. In this case, the patient presented with a Type I fusion between C2 and C3, which is consistent with the vertebral anomaly most frequently observed in KFS.

Maurice Klippel and André Feil classified three types of ankylosing spondylitis based on the extent and location of vertebral fusion, as well as associated vertebral abnormalities.

• Type I: massive fusion of cervical and upper thoracic vertebrae.
• Type II: fusion of two or more vertebrae with atlanto-occipital assimilation, hemivertebrae, or other cervical spine anomalies.
• Type III: Fused cervical vertebrae with lower thoracic or lumbar vertebrae.

The confusion stemming from the previous classification system provided sufficient impetus to justify developing a new system. In 1998, Clarke et al. developed a new system that delineates four classes of KFS based on etiology and genetic origins. Family-based studies of KFS have highlighted significant correlations between the location of the most rostral fusion, inheritance patterns, and specific anomalies associated with the syndrome.

• KF1 ± C1 fusion is not dominant, yet it is the most rostral fusion recorded in this class. There are often severe associated anomalies, including a very short neck. Fusion is obvious at birth. The mode of inheritance is always recessive.
• KF2 ± C2 ± 3 fusion is dominant. C2 ± 3 fusion is always the most rostral and is apparent postnatally. This is a dominant mode of inheritance.
• KF3 ± C3 (C2 ± 3 or C3 ± 4) fusion is not dominant, but it is the most rostral fusion recorded in this class. There is often a single, isolated fusion. Reduced penetrance inheritance.
• KF4±: Vertebral fusion and ocular anomalies are associated with Wildervanck syndrome. Possible X-linked inheritance [11].

The most recent and widely adopted classification was proposed by Samartzis et al. in 2006. This system is based on radiological findings and distinguishes three types, ranging from mildest to most severe:

• Type I, characterized by a single congenitally fused cervical segment;
• Type II, involving multiple noncontiguous congenitally fused segments;
• Type III, defined by multiple contiguous congenitally fused segments [12].

Our patient had a C2-C3 vertebral fusion and would therefore be classified as having Type I KFS, according to the classification system proposed by Samartzis et al.

Spinal fusion complications are common and can lead to various issues, including accelerated degeneration of adjacent spinal levels. This degeneration often manifests as disc degeneration, fractures, spondylosis, spinal canal stenosis, and osteophyte formation. These issues can ultimately result in spinal cord compression [[13], [14], [15]].

Patients with Type III KFS are typically at an increased risk for neurological symptoms and complications requiring surgical intervention; however, this was not the case for our patient.

The onset of neurological symptoms varies depending on the level of cervical fusion. Fusions at the C1-C2 level are typically associated with symptom manifestation in the first decade of life, whereas C2-C3 fusions are more commonly linked to symptom onset in the third decade. However, other reports suggest that the specific level of vertebral fusion may not significantly influence the overall incidence of neurological symptoms [16].

Significant central cord myelopathy has been reported in patients with KFS following moderate or even minor trauma. This is likely due to altered biomechanics caused by fused cervical segments, which increase mobility and stress adjacent, nonfused segments [16].

The differential diagnosis of KFS includes various congenital and acquired conditions that cause cervical vertebral abnormalities. Syndromes such as VACTERL, Goldenhar, and Wildervanck are often associated with multisystem anomalies, including renal, cardiac, auditory, and craniofacial defects [17,18].

In older children and adults, conditions such as resolving osteomyelitis or discitis, prior spinal fusion without instrumentation, juvenile idiopathic arthritis, rheumatoid arthritis, and ankylosing spondylitis should also be considered. These disorders may mimic atypical presentations of KFS with symptoms such as cervical pain, limited mobility, and vertebral shortening.

An accurate diagnosis requires a thorough clinical evaluation and appropriate imaging studies [6]. For instance, Čota et al. [12] reported a case of KFS that was initially misdiagnosed as spondyloarthropathy, which illustrates the diagnostic challenge. However, our case showed a classic Type I KFS presentation without any diagnostic confusion.

In terms of management, the primary focus of a treatment protocol for KFS is alleviating pain and tenderness. According to a recent study, most patients are treated conservatively, except in cases involving acute neurological deficits, cervical instability, or chronic neurological issues that pose significant risks and require surgical intervention [6].

In addition to surgery for specific skeletal and extraskeletal abnormalities when indicated, intensive physical therapy has been shown to improve strength and mobility. This therapy includes active range-of-motion exercises for the cervical spine and upper extremities, scapular retraction, and sternocleidomastoid muscle stretching.

Pharmacological treatments, including antidepressants, nonsteroidal anti-inflammatory drugs, corticosteroids, and muscle relaxants, may also be employed.

In our case, we guided the patient in kinesthetic awareness techniques to maintain proper posture and taught her breathing exercises to enhance lung capacity. We also discussed preventive strategies with her parents to avoid secondary complications. Emphasizing kinaesthetic awareness promoted correct posture, and regular physical therapy sessions helped establish a consistent exercise routine, ultimately improving her overall well-being.

## Conclusion

KFS individuals often experience neck and back pain, breathing difficulties, and limited mobility. In severe cases, they may also face a higher risk of mortality due to delayed detection. Proper precautions and close follow-up are therefore essential, especially for individuals with Type III disease.

Since symptoms vary from case to case, treatment and management must be tailored accordingly. Nevertheless, recent guidelines emphasize the importance of a multidisciplinary approach to care, especially for pediatric patients.

## Patient consent

Written informed consent was obtained from the patient for the publication of this case report.
